# Therapeutic Effect of* Cucumis melo* L. Extract on Insulin Resistance and the Gut Microbiome in Lep^ob^/Lep^ob^ Mice

**DOI:** 10.1155/2018/8159261

**Published:** 2018-02-05

**Authors:** Daeun Lee, Jung Hwa Yoo, Byung-Cheol Lee

**Affiliations:** Department of Clinical Korean Medicine, Graduate School, Kyung Hee University, 26 Kyungheedae-ro, Dongdaemun-gu, Seoul 02447, Republic of Korea

## Abstract

Obesity results in the progression of metabolic disorders, especially type 2 diabetes (T2DM). Obesity-induced insulin resistance (IR) is a causative factor of T2DM morbidity in obese people. It is generally held by clinicians that IR is caused by adiposity-related inflammation that is mediated by changes in composite ions in the gut microbiome. This experimental study was designed to investigate the effects of* Cucumis melo *L. (Cucumis) on obesity-induced IR in genetically leptin-deficient Lep^ob^/Lep^ob^ mice. Specifically, we examined the anti-inflammatory effects of Cucumis and the effects of Cucumis on the gut microbiota. We evaluated glucose control by measuring FBS, performing the OGTT, quantifying serum IR, calculating the HOMA-IR, and determining the lipid profiles. To see whether inflammation was reduced, we analyzed adipose tissue macrophages as well as monocytes in the blood. We also profiled the gut microbiota to determine whether the ratios of microbial phyla changed. We found that Cucumis improved IR in obese mice and relieved inflammation in adipose tissue and blood. Simultaneously, the microbiota composition ratios changed. In conclusion, administration of Cucumis improved IR by reducing inflammation, thereby changing the gut microbiota composition. Cucumis is thus a promising treatment for obesity-induced insulin resistance and the inflammatory state.

## 1. Introduction

Obesity is associated with an increased incidence of metabolic diseases including type 2 diabetes (T2DM), cardiovascular disease, and various cancers [1, 2]. Moreover, the prevalence of obesity is increasing. Obesity also results in the progression of T2DM through deterioration of obesity-induced insulin resistance (IR), which is an inability of cells to respond to insulin properly. Obesity impedes the regulation of glucose control by disturbing insulin signaling transduction, which has been shown to be affected by the gut microbiome [3]. It has been widely suggested that certain types of gut microbiota increase metabolic endotoxin secretion, especially lipopolysaccharide (LPS). These endotoxins lead to chronic inflammation, resulting in obesity and IR [3, 4]. Several studies have indicated that chronic inflammation is the underlying pathogenic mediator of insulin resistance [5–7]. The microbiota composition is influenced by several factors, such as changes in diet and medication use. Recently, worldwide efforts have focused on developing new medicines targeting the gut microbiota for treating obesity and prediabetes.

The stalk end of* Cucumis melo *L. (hereafter referred to as Cucumis), commonly known as muskmelon, is an herbal medicine that has been used to relieve congested Qi in the body and eliminate unnecessary body fluids. Although Cucumis is widely used in clinical situations, the effects of Cucumis have only been addressed in a few studies of emotional disorder [8] and facial pain [9]. Since Cucumis is used for its ability to affect the gastrointestinal tract and induce diarrhea, Cucumis is expected to affect the gut microbiota constituents, leading to improved glycemic control. Cucumis has been used in patients with metabolic diseases, especially obesity with insulin resistance. One clinical trial reported that Cucumis exerted therapeutic effects on fasting glucose level in patients with diabetes [10]. Another study showed that Cucumis improved insulin resistance and the gut microbiome in a mouse model [11]. However, the exact mechanism by which Cucumis exerts these effects has not yet been established. The main aim of this study was to determine the effectiveness of Cucumis in overcoming obesity-induced impaired glycemic control. A second aim was to determine the mechanism by which Cucumis exerts its effects by evaluating the levels of inflammation and gut microbiota composition in mice.

## 2. Materials and Methods

### 2.1. Preparation of* Cucumis melo* L.

*Cucumis melo *L. was purchased from the Department of Pharmaceutical Preparations at the Hospital of Korean Medicine, Kyung Hee University (Seoul, South Korea). Extractions were prepared after 100 g of dried Cucumis was added to 1,500 ml of 80% ethanol and boiled for 2 hours with a heating mantle. The sieve-filtered solution was filtered using an applicator and a 500 ml flask, after which it was concentrated with a rotary evaporator (model NE-1, EYELA Co., Japan). The solution was freeze-dried, and the extract was stored at room temperature. The final yield was 19.28%.

### 2.2. Animals and Experimental Design

Five normal (normal, *n* = 5) 6-week-old C57BL/7 mice weighing 19–21 g (Central Lab Animals Inc., Korea) and 10 obese 6-week-old C57BL/6 Lep^ob^/Lep^ob^ mice weighing 28–32 g (Central Lab Animals Inc., Korea) were used. The obese mice were genetically engineered to be leptin-deficient. The 10 ob/ob mice were randomly assigned to two groups: ob/ob (control, *n* = 5) or ob/ob treated with* Cucumis melo *L. (Cucumis, *n* = 5). Only male animals were used. The animal room was maintained at 40–70% humidity under a standard light cycle (12 h light/dark). Mice were given ad libitum access to food and water. After an observational period of one week, Cucumis extract (20–40 mg/kg) was administered* p.o.* twice a week to mice in the Cucumis group, while mice in the normal and control groups received normal saline. All animal work was authorized by the Kyung Hee Medical Animal Research Ethics Committee (KHMC-IACUC 14-025).

### 2.3. Metabolic Phenotype Measurements

#### 2.3.1. Weight Measurement (Body Weight, Food Intake, and Epididymal Fat)

Body weight was recorded at the beginning of the experiment, at the end of the experiment, and at weekly intervals. Weight was recorded using an electronic scale (CAS 2.5D, Seoul, Korea). Animals were weighed at the same time in the morning in order to minimize error. The amount of food that mice in each group consumed was measured weekly using the same scale. After mice were sacrificed at week 8, all epididymal fat pads were excised immediately and weighed.

#### 2.3.2. Oral Glucose Tolerance Test

Fasting blood glucose (FBG) tests were performed at baseline and at 8 weeks. Mice were fasted for 6 hours prior to each test. After fasting, glucose level was measured from the tail vein with an ACCU-CHECK Performa glucose meter. The OGTT was performed after 14 h of overnight fasting. Body weights were measured before the test to determine the oral glucose load in each mouse (2.0 g/kg body weight). Blood samples were collected from the tail vein before administration and at 30, 60, 90, and 120 min after administration.

#### 2.3.3. Fasting Insulin Concentration and Lipid Profile Analysis

Mice were fasted overnight and then sacrificed, after which blood samples were immediately obtained by heart puncture. The blood samples were centrifuged at 3,000 rpm for 20 min to obtain serum. Fasting insulin level was assessed using an ELISA kit (Crystal Chem). Insulin standards and samples were aliquoted into antibody-coated microplates, after which they were mixed with an anti-insulin enzyme conjugate for 30 min at room temperature. After 7 washes, the reactions were incubated for 40 min with enzyme substrate solution. After adding the stopping solution to the reaction and incubating for 10 min, the absorbances at 450 nM were determined using an ELISA reader. The HOMA-IR value was calculated based on the FBG and insulin levels [12]. Total serum, HDL, LDL, NEFA, and TG levels were measured from the blood samples.

#### 2.3.4. Hepatic and Renal Function Safety Test

After serum was obtained by centrifugation, the supernatant was frozen at −40°C until analysis. Aspartate aminotransaminase (AST), alanine aminotransaminase (ALT), and creatinine levels were measured.

### 2.4. Adipose Tissue Macrophage (ATM) Gating and Monocyte Gating

#### 2.4.1. Stromal Vascular Cell (SVC) Segregation

Stromal vascular cells (SVCs) were isolated from samples using a well-established collagenase-based method [13, 14]. In brief, harvested epididymal fat pads were soaked in PBS (Gibco) and 2% BSA (Gibco), after which they were pulverized into pieces 1-2 mm in diameter. Next, collagenase (Sigma) and DNase I (Roche) were added to the samples, after which the reactions were shaken for 20 min at 37°C with the addition of 2% BSA/PBS and 5 mM EDTA. After filtration through 100 *μ*M nylon mesh (BD Science), the mixtures were centrifuged at 1000 rpm for 3 min to remove the supernatants. The pellets at the bottom were mixed with PBS and 2% FBS (Sigma). The samples were filtered again using the 100 *μ*m nylon mesh to remove unnecessary tissue. After centrifugation at 200 rpm for 10 min, SVCs were obtained from the bottom of the samples.

#### 2.4.2. Fluorescence Activated Cell Sorting (FACS) Analysis of ATMs and Monocytes

The numbers of SVCs in adipose tissue and blood samples in 5 mM EDTA were determined using a cellometer (Nexcelom Bioscience LLC). Samples were incubated for 10 min with BD Fc Block (BC Pharmingen) at a ratio of 1 : 100. Fluorophore-conjugated antibodies were added, and the mixtures were incubated in the dark for 20 min. A FACSCalibur (BD Biosciences) instrument was used to analyze the samples. The following antibodies were used for staining: CD45-APC Cy7 (BioLegend), CD68-APC (BioLegend), CD11c-phycoerythrin (BioLegend), CD206-FITC (BioLegend), CD45-APC Cy5.5 (BioLegend), and CD11b-PE (BioLegend). The percentage of cells stained with each was further analyzed using FlowJo (Tree Star).

### 2.5. 16s Ribosomal RNA Sequencing Analysis of the Gut Microbiota

The entire 16s ribosomal RNA sequencing process was performed by Macrogen Inc. using Illumina technology. The Illumina NGS workflow includes 4 steps: sample preparation, library construction, sequencing, and raw data. Sample preparation corresponds to extraction of DNA/RNA and quality control analysis. After preparation, the library is constructed by random fragmentation of the DNA or cDNA sample, followed by 5′ and 3′ adapter ligation. Adapter-ligated fragments are PCR-amplified and gel-purified. PCR is used to amplify the template of a DNA sample using template-specific primers with attached overhand adapters. Reactions consisted of 2.5 ul microbial DNA, 12.5 ul 2x KAPA HiFi HotStart ReadyMix, and 5 ul of each primer (1 uM, forward and reverse). The sample is sealed, and PCR is performed in a thermal cycler. After amplification, PCR clean-up is performed. The PCR plate is centrifuged at 1,000 ×g at 20°C for 1 min to collect condensation and remove the seal.

For cluster generation, the library is loaded into a flow cell where fragments are captured on a lawn of surface-bound oligos complementary to the library adapters. Each fragment is then amplified into distinct, clonal clusters through bridge amplification. When cluster generation is complete, the templates are ready for sequencing. Illumina SBS technology utilizes a proprietary reversible terminator-based method that detects single bases as they are incorporated into DNA template strands. Since all 4 reversible, terminator-bound dNTPs are present during each sequencing cycle, natural competition minimizes incorporation bias. Sequencing data is then converted into raw data for the analysis.

### 2.6. Statistical Analysis

Results were statistically analyzed by GraphPad Prism 7 (GraphPad Software, Inc.). Groups were compared using one-way ANOVA and Tukey's post hoc test. Data are presented as mean ± SEM; two-tailed *p* values were regarded as significant if <0.05.

## 3. Results

### 3.1. Effects of* Cucumis melo* L. on Body Weight, Epididymal Fat Weight, and Food Consumption

**Figure 1:**
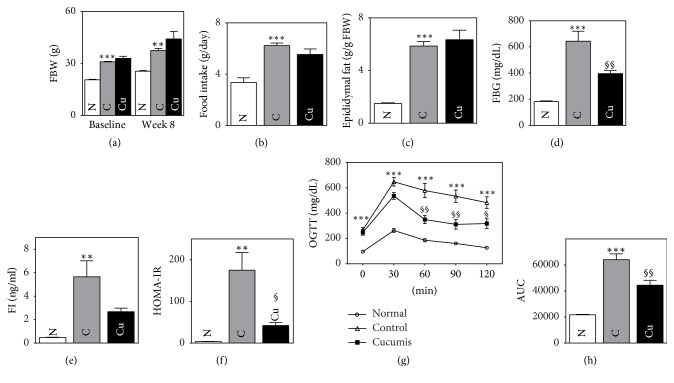
Cucumis p.o. administration improves glycemic control and insulin resistance in* C57BL/6 *Lep^ob^/Lep^ob^ mice. (a) Fasting body weight at baseline and at week 8. (b) Food intake per day during the experimental study. (c) Epididymal fat weight at week 8. (d) Fasting blood glucose levels. (e) Fasting serum insulin levels. (f) HOMA-IR. (g) Oral glucose tolerance test. (h) Area under the curve (AUC) of the OGTT.* Data are presented as mean ± SEM. *^§^*p* < 0.05, ^*∗∗*,§§^*p* < 0.01, and ^*∗∗∗*^*p* < 0.001.

Control mice had a higher average fasting body weight than normal mice, both at the beginning of the experiment and at week 8 (29.12 ± 0.28 versus 20.77 ± 0.38 g, *p* < 0.001, and 37.41 ± 2.79 versus 25.52 ± 0.37 g, *p* < 0.01, resp.). However, the control mice did not have a significantly higher weight gain between these two time points. There was no significant difference in the weights of obese mice or in the weight gains between baseline and week 8. Control mice had significantly heavier epididymal fat pads compared to normal mice (2.21 ± 0.28 g versus 0.38 ± 0.03 g, *p* < 0.001). However, fat weights were not significantly different between the control and Cucumis groups. Food consumption per day was higher in the control group compared to the normal group (6.22 ± 0.21 versus 3.34 ± 0.37 g, *p* < 0.001). However, mice in the Cucumis group did not consume more food than mice in the control group (Figures 1(a)–1(c)).

### 3.2. Effect of* Cucumis melo *L. on Glucose Control

At baseline, control mice had a significantly higher FBG level compared to that of normal mice (298.8 ± 12.5 versus 170.2 ± 6.0 mg/dl, *p* < 0.01). While there was no difference between the control and Cucumis groups at baseline, the Cucumis group had a significantly lower FBG level compared to the control group at week 8 (519.0 ± 50.5 versus 277.7 ± 20.7 mg/dl, *p* < 0.01). All groups had their highest glucose levels at the 30 min time point (Figure 1(g)). Moreover, the control group had higher glucose levels for all points than the normal group; these differences were all significant (all *p* < 0.001). The glucose levels of the Cucumis group were significantly lower than those of the control group for all time points, except for the 0 and 30 min time points. The area under the curve (AUC) of the control group was significantly higher than that of the normal group (21669 ± 350 versus 64023 ± 4463 mg·min/dL, *p* < 0.001), and the Cucumis group showed a significantly lower AUC compared to the control group (64023 ± 4463 versus 44379 ± 3214 mg·min/dL, *p* < 0.01). These data are shown in Figures 1(d), 1(g), and 1(h).

### 3.3. Effect of* Cucumis melo *L. on Insulin Resistance

Insulin resistance was evaluated by calculating the HOMA-IR values. While the control group had a higher HOMA-IR value than the normal group (3.72 ± 0.21 versus 175.13 ± 42.40, *p* < 0.01), the Cucumis group had a significantly lower HOMA-IR value than the control group (42.03 ± 5.40 versus 175.13 ± 42.40, *p* < 0.05). Fasting insulin concentration (FI) was significantly increased in the control group compared to the normal group (5.66 ± 1.36 versus 0.47 ± 0.03 ng/ml, *p* < 0.01). Although the FI of the Cucumis group was lower than that of the control group, this difference was not significant (2.67 ± 0.24 versus 5.66 ± 1.36 ng/ml). These data are shown in Figures 1(e) and 1(f).

### 3.4. Effects of* Cucumis melo *L. on Lipid Profiles

**Figure 2:**
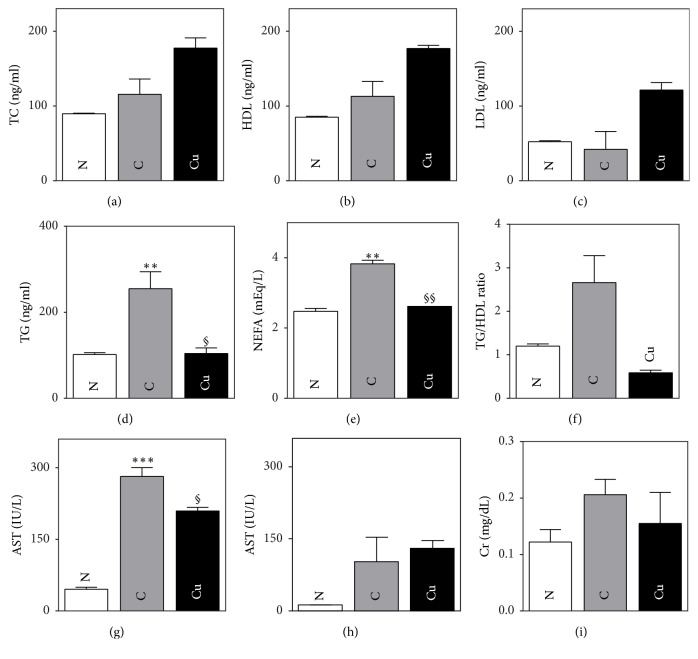
Cucumis p.o. administration improves lipid profiles in C57BL/6 *Lep*^*ob*^/*Lep*^*ob*^ mice and shows a safe hepatic and renal profile. (a–c) Total cholesterol, high-density lipoprotein cholesterol, and low-density lipoprotein cholesterol levels. (d) Triglyceride levels. (e) Nonesterified fatty acid levels. (f) Ratios of triglycerides to high-density lipoprotein cholesterol. (g-h) Aspartate aminotransaminase and alanine aminotransaminase levels. (i) Creatinine levels.* Data are presented as mean ± SEM*. ^§^*p* < 0.05,^*∗∗*,§§^*p* < 0.01, and ^*∗∗∗*^*p* < 0.001.

There were no significant differences between any of the groups with respect to total cholesterol, HDL, or LDL-C. However, the control group had significantly elevated levels of TG and NEFA compared to the normal group. The levels of TG and NEFA were significantly decreased in the Cucumis group (254.60 ± 39.66 versus 80.89 ± 31.65, *p* < 0.05, and 3.83 ± 0.10 versus 2.61 ± 0.06, *p* < 0.001, resp.). These data are shown in Figures 2(a)–2(f).

### 3.5. Effects of* Cucumis melo *L. on Hepatic and Kidney Safety

To confirm the safety of Cucumis, serum AST, ALT, and creatinine levels were analyzed. AST was significantly elevated in the control group compared to the normal group (281.80 ± 18.44 versus 45.60 ± 3.96 IU/L, *p* < 0.001). On the other hand, ALT levels were not significantly different between the normal and control groups. Regarding renal function, the control group had a significantly elevated creatinine level compared to the normal group (0.21 ± 0.03 versus 0.12 ± 0.02 mg/dl *p* < 0.05). The Cucumis group had a lower level of AST (281.80 ± 0.55 versus 103.20 ± 50.35 IU/L, *p* < 0.05) compared to the control group. There were no significant differences in creatinine or ALT between any of the groups. These data are displayed in Figures 2(g)–2(i).

### 3.6. Effect of* Cucumis melo* L. on Inflammation

#### 3.6.1. Adipose Tissue Macrophages

**Figure 3:**
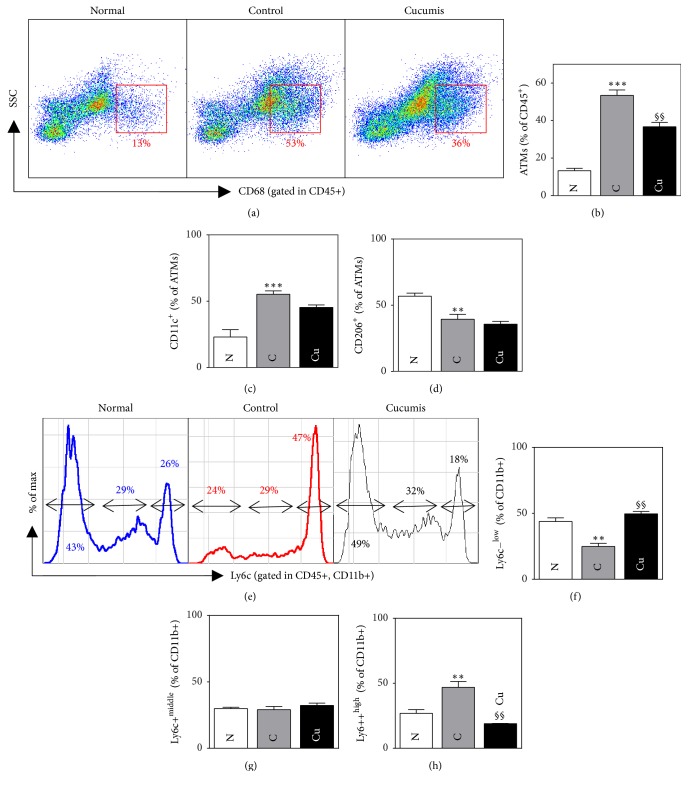
Cucumis p.o. administration reduces inflammation in C57BL/6 Lep^ob^/Lep^ob^ mice. (a) Flow cytometry results showing adipose tissue macrophage gating. (b) Percentages of adipose tissue macrophages. (c-d) Percentages of CD11c^+^ and CD206^+^ adipose tissue macrophages. (e) Monocyte infiltration rate. (f–h) Percentages of Ly6c^−^, Ly6c^+^, and Ly6c^++^ cells in the CD11b+ population.* Data are presented as mean ± SEM*. ^*∗∗*,§§^*p* < 0.01, and ^*∗∗∗*^*p* < 0.001.

The total percentage of CD45+ macrophages in adipose tissue was significantly higher in the control group compared to the normal group (53.66 ± 2.91 versus 13.23 ± 1.25%, *p* < 0.001). Compared to the control group, the Cucumis group showed a significantly smaller percentage of macrophages (36.52 ± 1.85 versus 53.66 ± 2.91%, *p* < 0.01). Moreover, the percentage of CD11+ macrophages was significantly higher in the control group compared to the normal group (55.23 ± 2.71 versus 22.75 ± 5.54%, *p* < 0.001). The Cucumis group, on the other hand, had a lower percentage of CD11+ macrophages compared to the control group, but this difference was not statistically significant. Regarding CD206+ macrophages, the control group had a significantly lower percentage than the normal group (39.49 ± 2.4 versus 56.73 ± 2.34%, *p* < 0.01). The Cucumis group also had a lower level of CD206+ macrophages; however, this difference was not significant. These data are presented in Figures 3(a)–3(d).

#### 3.6.2. Blood Monocytes

The percentage of Ly6c^hi^ monocytes was significantly higher in the control group than in the normal group (46.87 ± 4.51 versus 26.89 ± 2.88%, *p* < 0.01), whereas the percentage of Ly6c^low^ monocytes was significantly lower in the control group than in the normal group (43.78 ± 2.80 versus 24.81 ± 2.44%, *p* < 0.01). Compared to the control group, the Cucumis group had a lower percentage of Ly6c^hi^ monocytes (18.94 ± 0.08 versus 46.87 ± 4.51%, *p* < 0.01) and a higher percentage of Ly6c^low^ monocytes (49.58 ± 1.13 versus 24.81 ± 2.44%, *p* < 0.01); both of these differences were significant. The percentages of Ly6c^middle^ monocytes were not significantly different between any of the groups. These data are presented in Figures 3(e)–3(h).

### 3.7. Effect of* Cucumis melo* L. on the Gut Microbiota

**Figure 4:**
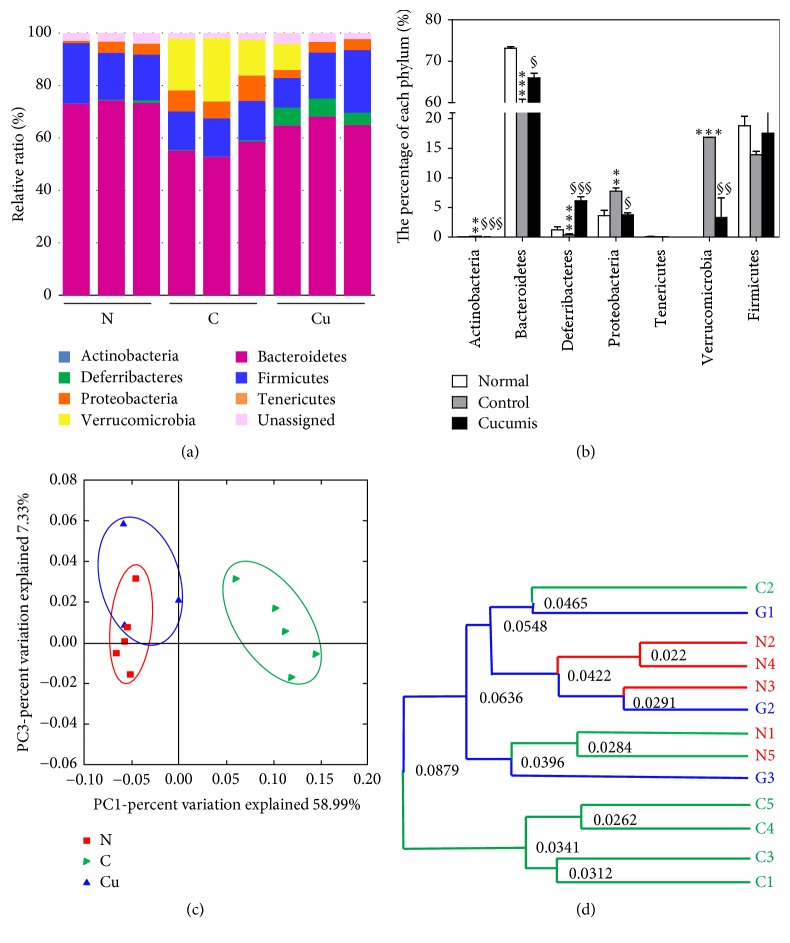
The gut microbiota proportions show favorable obesity-related changes in the Cucumis-treated group. (a) Percentage of each phylum in the gut microbiota. (b) Relative ratios of phyla in a single individual. (c) Two-dimensional principal coordinate analysis (2D PCoA) of individuals in each group. (d) Unweighted pair group method with arithmetic mean (UPGMA) tree.* Data are presented as mean ± SEM*. ^§^*p* < 0.05,^*∗∗*,§§^*p* < 0.01, and^*∗∗∗*,§§§^*p* < 0.001.

Significantly different proportions of gut microbiota constituents were observed between the control and normal groups; specifically, Actinobacteria and Verrucomicrobia were significantly more abundant in the control group (0.16 ± 0.02 versus 0.06 ± 0.01%, *p* < 0.01, and 0.00 ± 0.00 versus 16.85 ± 2.43%, *p* < 0.001). The control group also exhibited a higher proportion of Proteobacteria (3.61 ± 0.91 versus 7.76 ± 0.53%, *p* < 0.01) and a significantly lower proportion of Bacteroidetes (73.13 ± 0.42 versus 58.49 ± 2.39%, *p* < 0.001). Compared to the control group, the Cucumis group showed significantly decreased percentages of Actinobacteria and Verrucomicrobia (0.16 ± 0.02 versus 0.02 ± 0.01%, *p* < 0.001, and 16.85 ± 2.43 versus 3.31 ± 3.31%, *p* < 0.01). In addition, the Cucumis group exhibited a significantly higher percentage of Bacteroidetes and a significantly lower proportion of Proteobacteria (58.49 ± 2.39% versus 64.12 ± 1.11%, *p* < 0.05, and 7.76 ± 0.53 versus 3.74 ± 0.36%, *p* < 0.05). The proportions of* Firmicutes *were not significantly different between any of the groups. The proportion of Deferribacteres was significantly increased in the Cucumis group. The proportions are listed in Table 1 and Figures 4(a) and 4(b); a visualization of the microbiota and a hierarchical clustering diagram are displayed in Figures 4(c) and 4(d).

## 4. Discussion

Obesity has become prevalent worldwide and is a risk factor for many chronic diseases, especially type 2 diabetes (T2DM) [15]. T2DM, the most common complication of obesity, is a complex illness requiring continuous care beyond glycemic control [16]. Obesity-induced insulin resistance (IR) is a primary component in the pathology of T2DM, meaning that improving IR is a promising strategy for preventing diabetes. The gut microbiota has recently been reported to disturb insulin signaling, which interferes with glucose regulation in obesity. This finding suggests that alterations in the gut microbiota in obesity lead to chronic inflammation, causing IR and eventually diabetes [4]. The stalk end of* Cucumis melo *L. (also referred to as Cucumis) has been reported to have a therapeutic effect on glucose control in patients with diabetes [10, 11]. Previous phytochemical studies have revealed* Cucumis melo* to be a rich source of volatile compounds, triterpenoids, sterols, and flavonoids [17], among which cucurbitacins have pharmacological effects, such as hepatoprotective, cardiovascular, purgative, antimicrobial, and anti-inflammatory activities [18].

In this experimental study, the two groups of leptin-deficient obese mice had similar baseline fasting blood glucose (FBG) levels. However, the Cucumis group had a lower FBG level at the 8-week time point compared to the control group. The OGTT can be used to identify individuals who have not yet been diagnosed with diabetes but who have prediabetes. The Cucumis group exhibited lower glucose levels at all time points of the OGTT compared to the control group, leading to a smaller AUC. This finding is suggestive of improved glycometabolism.

The most widely accepted proposed mechanism for how the gut microbiome worsens obesity and IR involves the impact of metabolic endotoxemia, specifically from microorganism-derived lipopolysaccharide (LPS). The LPS endotoxin is secreted from certain bacteria and has been shown to contribute to chronic underlying inflammation, thus driving the development of adiposity and IR [19]. Our findings are consistent with this mechanism, since the Cucumis group showed improved inflammatory status. The microbiota constituents and levels of inflammation were analyzed to test the hypothesized mechanism of the effects of Cucumis. Among the hundreds of microorganisms in the gut, the predominant phylum that has been linked to adiposity is Bacteroidetes [16], the most abundant phylum in the gut. Specifically, the proportion of Bacteroidetes has been shown to be decreased in obese individuals relative to lean individuals [20]. This proportion trend was also seen in our results; that is, the proportion of Bacteroidetes was reduced in control mice compared to normal mice. However, Cucumis administration significantly increased the percentage of Bacteroidetes relative to that of the control group. Another phylum discussed in the context of obesity is Firmicutes, the second most abundant phylum. In contrast to Bacteroidetes, the ratio of Firmicutes has been shown to be increased in obese individuals in several studies [2, 20]. However, unlike Bacteroidetes, which has been shown to be clearly decreased in obese individuals in most studies, it is still debated whether the proportion of Firmicutes is elevated in obese individuals. For instance, some studies did not find a significant difference in the proportion of Firmicutes between lean and obese individuals [21]. Our experimental study contributes information to this debate about the role of Firmicutes in obesity. We found that the proportion of Firmicutes was not elevated in the control group and was not altered by Cucumis treatment. In addition to these two phyla, the Actinobacteria ratio has been demonstrated to be altered in overweight individuals. In a microbiome study, the Actinobacteria proportion in the gut was shown to be increased in both genetically obese and high-fat-diet-induced obese mice [21]. Similarly, the proportion was reported to be higher in obese human adults than in the lean individuals [20]. We found that the Actinobacteria ratio was higher in the control group compared to the normal group and that Cucumis treatment decreased this proportion; these differences were all statistically significant. This finding agrees with the previous discovery that the phylum Actinobacteria is one of the most abundant phyla in the gut environment of obese individuals.

Chronic inflammation mainly originates from adipose tissue [22]. Adipose tissue macrophages (ATMs) correlate with increased production of inflammatory molecules and are therefore a crucial factor in inflammation [7]. Macrophages exhibit either a proinflammatory or an anti-inflammatory phenotype and are classified into classically activated M1 and alternatively activated M2 macrophages. M1 macrophages secrete high levels of proinflammatory cytokines, such as tumor necrosis factor-*α* (TNF-*α*), interleukin-6 (IL-6), and IL-1*β*, and reactive oxygen species, causing inflammatory reactions. On the other hand, M2 macrophages secrete anti-inflammatory cytokines such as IL-10, transforming growth factor-*β* (TGF-*β*), and IL-4, thus blocking processes mediated by M1 macrophages [23]. We found that* p.o.* administration of Cucumis resulted in improved levels of inflammation with a significantly reduced total percentage of ATMs and a lower percentage of CD11C+ M1 macrophages, which also showed an increased percentage of CD206 M2 macrophages. Although levels of cytokines were not evaluated in this study, the lowered percentage of M1 macrophages obviously supposes the reduced level of inflammatory cytokines of TNF-*α*, IL-6, and IL-1*β* and an increased percentage of CD206+ M2 macrophages also suggesting the increased level of anti-inflammatory cytokines of IL-10, TGF-*β*, and IL-4. The tendency of decreased and elevated proportions of CD11+ and CD206 ATMs, respectively, might reflect a trend of cytokine expression being driven from M1 to M2 macrophages, thus ameliorating the inflammatory status in adipose tissue.

Monocytes are phagocytic cells generated in the bone marrow and released into the bloodstream. They are present in at least two distinct populations in mice: classical “proinflammatory” cells expressing high levels of Ly6C and nonclassical “patrolling” alternative monocytes with low levels of Ly6C or no Ly6C at all [24]. Ly6C^hi^ monocytes circulating in the blood respond to inflammatory lesions, to which they are recruited, where they differentiate into inflammatory macrophages. On the other hand, in the absence of inflammation, Ly6C^low^ monocytes enter normal tissues and replenish the tissue-resident macrophages [25]. Compared to control mice, Cucumis-administrated mice exhibited a significantly smaller percentage of Ly6C^hi^ monocytes. In this study, levels of serum cytokines in each group were not analyzed; however, as Ly6C^hi^ monocytes secrete inflammatory cytokines of TNF-*α* and IL-1*β* [26], it can be perceived that inflammatory cytokines diminish in level as well. On the other hand, a percentage of Ly6C^low^ which produce an anti-inflammatory cytokine IL-10 [27] increased significantly in the Cucumis-treated group, suggesting an elevated level of IL-10. This finding indicates that Cucumis treatment lowered the percentage of monocytes in the blood with inflammatory tendencies but increased the percentage of monocytes in the blood with anti-inflammatory tendencies. This outcome is interesting as it shows that Cucumis has an effect on monocytes in the bloodstream, indicating systemic effects, as well as its local effects on adipose tissue-resident macrophages as mentioned earlier.

The Cucumis group exhibited reduced insulin levels and HOMA-IR scores, both of which were reduced to a large degree; however, this difference was only significant for HOMA-IR. In lipolysis, NEFAs are released from TG. This process has been shown to be sensitive to suppression by insulin [15]. In addition, NEFAs influence glucose transport, and several studies have revealed a relationship (the “glucose fatty acid cycle”) among elevated NEFA level, insulin resistance (IR), and development of diabetes [28, 29]. Therefore, the elevated NEFA levels in the control group suggest that the control animals were deficient in their ability to use insulin, indicating the presence of IR. In contrast, the significantly reduced level of NEFAs in the Cucumis group means that these animals used insulin more efficiently than control animals. Since insulin is necessary to remove TG from the bloodstream, high TGs are one of the first symptoms of IR in prediabetes. Of note, Cucumis administration significantly reduced the level of TG compared to the level in the control group. The reduced TG and NEFA levels in the Cucumis-treated group suggest improved IR. The ratio of TG to HDL cholesterol is a clinical indicator of IR, with a higher value signifying aggravated IR [30]. We found that the TG-to-HDL ratio in the Cucumis group was much lower than the ratios in the other two groups, although these differences were not significant.

Cucumis is used to purposely induce diarrhea to treat several diseases; therefore, we evaluated liver and kidney function by measuring aminotransferase and creatinine levels. The AST, ALT, and creatinine levels were not increased in the Cucumis group, indicating that Cucumis is safe and did not have liver or kidney toxicity. This outcome is consistent with that of a retrospective study [31] investigating the safety of Cucumis in humans. The AST level was even significantly lower in the Cucumis group compared to the control group. However, since Cucumis is regarded as the most powerful treatment in Korean medicine, it should only be used after carefully considered prescription by a doctor specializing in Korean medicine.

We found that administration of Cucumis ameliorated the impaired glucose control observed in obese mice by reducing inflammation. This was achieved by favorably changing the gut microbiota to release less LPS endotoxin. This result suggests that Cucumis is potentially a promising option for preventing obesity and diabetes, two inflammation-related metabolic diseases.

## 5. Conclusion

Oral administration of* Cucumis melo *L. to leptin-deficient obese mice resulted in improved inflammatory status associated with alteration of the gut microbiota, leading to better glycemic control. This finding suggests that* Cucumis melo *L. merits further investigation as a potential treatment for obesity-induced insulin resistance.

## Figures and Tables

**Table 1:** Gut microbiota composition of each group at the phylum level (%).

	Normal	Control	Cucumis
Actinobacteria	0.06 ± 0.01	0.16 ± 0.02^*∗∗*^	0.02 ± 0.01^§§§^
Bacteroidetes	73.13 ± 0.42	58.49 ± 2.39^*∗∗∗*^	64.12 ± 1.11^§^
Deferribacteres	1.21 ± 0.56	0.38 ± 0.18^*∗∗∗*^	6.12 ± 0.72^§§§^
Proteobacteria	3.61 ± 0.91	7.76 ± 0.53^*∗∗*^	3.74 ± 0.36^§^
Tenericutes	0.09 ± 0.05	0.03 ± 0.01	0.00 ± 0.00
Verrucomicrobia	0.00 ± 0.00	16.85 ± 2.43^*∗∗∗*^	3.31 ± 3.31^§§^
Firmicutes	18.79 ± 1.64	13.93 ± 0.55	17.57 ± 3.62

Data are presented as mean ± SEM. ^*∗*,§^*p* < 0.05, ^*∗∗*,§§^*p* < 0.01, and ^*∗∗∗*,§§§^*p* < 0.001.
